# Stepped-wedge cluster randomised controlled trials: some variations on the common design

**DOI:** 10.1186/1745-6215-14-S1-P15

**Published:** 2013-11-29

**Authors:** Karla Hemming, Alan Girling

**Affiliations:** 1 University of Birmingham, Birmingham, UK

## 

Stepped wedge cluster randomised trials (SW-CRTs) are being used with increasing frequency in health service evaluation. Conventionally, these studies are designed with equally spaced steps, with an equal number of clusters randomised at each step, and data collected at each and every step.

Here we introduce several variations on this design and consider implications for power. One modifiction we consider is where the number of clusters varies at each step, or where at some steps data are not collected. We show that the parallel CRT with staggered, but balanced randomisation, can be considered a special case of the incomplete SW-CRT. And, we extend these designs to allow for multiple layers of clustering, for example wards within a hospital. Building on results for complete designs, power and detectable difference are derived using Wald-test and obtaining the variance-covariance matrix of the treatment effect assuming a generalised linear mixed model. These variations are illustrated by several real examples.

